# Supplementary material for: How tp1, an indirect wing steering muscle, stabilizes Drosophila’s flight
Source: bioRxiv. 2025 Nov 8:2025.11.02.686144. Preprint. [Version 2] doi: 10.1101/2025.11.02.686144 (PMC12637562; doi:10.1101/2025.11.02.686144)
Supplement: Supplement 1 [file NIHPP2025.11.02.686144v2-supplement-1.pdf]

bioRxiv preprint doi: https://doi.org/10.1101/2025.11.02.686144; this version posted November 8, 2025. The copyright holder for this preprint (which was not certified by peer review) is the author/funder, who has granted bioRxiv a license to display the preprint in perpetuity. It is made available under aCC-BY 4.0 International license.

Supplementary Note 1: Supplementary Information

A. FANC Dataset: Premotor neurons from haltere tract innervating wing motoneurons

pre_pt_root_id	pref_mn	pref_module	classification_system	putative_NT	ps1	ps2	tp1	tp2	tpn	i1	i2	iii1	iii3	iii4	hg1	hg2	hg3	hg4	b1	b2	b3	MN_syn_total
648518346470508288	iii4a_u	steerD_syn	sensory	unk	0	0	0	0	0	0	0	0	0	16	7	0	0	0	0	11	0	34
648518346485536768	iii4a_u	steerD_syn	sensory	unk	0	0	0	0	0	0	0	0	0	20	7	0	0	0	0	5	0	32
648518346475410304	iii4a_u	steerD_syn	sensory	unk	0	0	0	0	0	0	0	0	0	11	0	0	0	0	0	8	0	19
648518346471718784	iii4a_u	steerC_syn	sensory	unk	0	0	0	0	0	0	0	0	0	6	0	0	0	0	6	5	0	17
648518346488013824	iii4a_u	steerD_syn	sensory	unk	0	0	0	0	0	0	0	0	0	10	0	0	0	0	0	4	0	14
648518346500991872	hg1_u	steerD_syn	sensory	unk	0	0	0	0	0	0	0	0	0	3	4	0	0	0	0	3	0	10
648518346486820224	hg4	steerD_syn	sensory	unk	0	0	0	0	0	0	0	0	0	12	0	0	0	14	0	10	0	36
648518346517916032	hg4	steerD_syn	sensory	unk	0	0	4	0	0	0	0	0	0	3	0	0	0	15	0	7	0	29
648518346486224512	hg4	steerD_syn	sensory	unk	0	0	0	0	0	0	0	0	0	0	0	0	0	24	0	3	0	27
648518346514248448	hg4	steerD_syn	sensory	unk	0	0	0	0	0	0	0	0	0	3	0	0	0	8	0	7	0	18
648518346492495232	hg4	steerD_syn	sensory	unk	0	0	0	0	0	0	0	0	0	0	0	0	0	10	0	6	0	16
648518346493760384	hg4	steerD_syn	sensory	unk	0	0	0	0	0	0	0	0	0	0	0	0	0	7	0	3	0	10
648518346491390464	b1	steerC_syn	sensory	unk	0	0	3	0	0	0	0	0	0	3	0	0	0	0	26	18	0	50
648518346499938816	b1	steerC_syn	sensory	unk	0	0	7	0	0	0	0	0	0	0	0	0	0	0	31	11	0	49
648518346494248448	b1	steerC_syn	sensory	unk	0	0	0	0	0	0	0	0	0	4	0	0	0	0	29	7	0	40
648518346496400000	b1	steerC_syn	sensory	unk	0	0	14	0	0	0	0	0	0	0	0	0	0	0	22	0	0	36
648518346515806208	b1	steerC_syn	sensory	unk	0	0	0	0	0	0	0	0	0	0	0	0	0	3	21	12	0	36
648518346480941440	b1	steerC_syn	sensory	unk	0	0	11	0	0	0	0	0	0	0	0	0	0	0	18	6	0	35
648518346474295936	b1	steerC_syn	sensory	unk	0	0	0	0	0	0	0	0	0	5	0	0	0	3	12	12	0	32
648518346492260480	b1	steerC_syn	sensory	unk	0	0	0	0	0	0	0	0	0	0	0	0	0	9	15	7	0	31
648518346496851840	b1	steerC_syn	sensory	unk	0	0	0	0	0	0	0	0	0	0	0	0	0	0	16	15	0	31
648518346466807936	b1	steerC_syn	sensory	unk	0	0	0	0	0	0	0	0	0	0	0	0	0	7	18	4	0	29
648518346501123840	b1	steerC_syn	sensory	unk	0	0	0	0	0	0	0	0	0	0	0	0	0	0	14	12	0	26
648518346484680320	b1	steerC_syn	sensory	unk	0	0	0	0	0	0	0	0	0	0	0	0	0	3	14	8	0	25
648518346486427264	b1	steerC_syn	sensory	unk	0	0	0	0	0	0	0	0	0	6	0	0	0	0	10	8	0	24
648518346502677248	b1	steerC_syn	sensory	unk	0	0	0	0	0	0	0	0	0	0	0	0	0	0	17	6	0	23
648518346494102656	b1	steerC_syn	sensory	unk	0	0	0	0	0	0	0	0	0	0	0	0	0	0	11	9	0	20
648518346491030016	b1	steerC_syn	sensory	unk	0	0	0	0	0	0	0	0	0	0	0	0	0	0	11	4	0	15
648518346507210624	b1	steerC_syn	sensory	unk	0	0	0	0	0	0	0	0	0	0	0	0	0	0	8	5	0	13
648518346517345024	b1	steerC_syn	sensory	unk	0	0	0	0	0	0	0	0	0	0	0	0	0	0	8	4	0	12
648518346488400000	b1	steerC_syn	sensory	unk	0	0	0	0	0	0	0	0	0	0	0	0	0	0	9	0	0	9
648518346477806080	b1	steerC_syn	sensory	unk	0	0	0	0	0	0	0	0	0	0	0	0	0	0	6	0	0	6
648518346509182720	b1	steerC_syn	sensory	unk	0	0	0	0	0	0	0	0	0	0	0	0	0	0	5	0	0	5
648518346484380928	b1	steerC_syn	sensory	unk	0	0	0	0	0	0	0	0	0	0	0	0	0	0	4	0	0	4
648518346478752384	b2	steerC_syn	sensory	unk	0	0	0	0	0	0	0	0	0	15	7	0	0	0	8	25	0	55
648518346465928448	b2	steerC_syn	sensory	unk	0	0	0	0	0	0	0	0	0	3	7	0	0	0	15	17	0	42
648518346486703360	b2	steerC_syn	sensory	unk	0	0	0	0	0	0	0	0	0	0	6	0	0	4	12	14	0	36
648518346492174976	b2	steerD_syn	sensory	unk	0	0	0	0	0	0	0	0	0	13	0	0	0	6	0	14	0	33
648518346481466368	b2	steerC_syn	sensory	unk	0	0	0	0	0	0	0	0	0	4	0	0	0	0	3	21	0	28
648518346515599616	b2	steerC_syn	sensory	unk	0	0	0	0	0	0	0	0	0	4	0	0	0	0	0	19	0	23
648518346514482688	b2	steerC_syn	sensory	unk	0	0	0	0	0	0	0	0	0	0	0	0	0	0	9	13	0	22
648518346488823040	b2	steerC_syn	sensory	unk	0	0	0	0	0	0	0	0	0	0	0	0	0	0	7	9	0	16

Figure S1. Premotor neurons from haltere tract innervating b1, b2 motoneurons

pre_pt_root_id	pref_mn	pref_module	classification_system	putative_NT	ps1	ps2	tp1	tp2	tpn	i1	i2	iii1	iii3	iii4	hg1	hg2	hg3	hg4	b1	b2	b3	MN_syn_total
648518346517916032	hg4	steerD_syn	sensory	unk	0	0	4	0	0	0	0	0	0	3	0	0	0	15	0	7	0	29
648518346491390464	b1	steerC_syn	sensory	unk	0	0	3	0	0	0	0	0	0	3	0	0	0	0	26	18	0	50
648518346499938816	b1	steerC_syn	sensory	unk	0	0	7	0	0	0	0	0	0	0	0	0	0	0	31	11	0	49
648518346496400000	b1	steerC_syn	sensory	unk	0	0	14	0	0	0	0	0	0	0	0	0	0	0	22	0	0	36
648518346480941440	b1	steerC_syn	sensory	unk	0	0	11	0	0	0	0	0	0	0	0	0	0	0	18	6	0	35

Figure S2. Premotor neurons from haltere tract innervating tp1 motoneuron

bioRxiv preprint doi: https://doi.org/10.1101/2025.11.02.686144; this version posted November 8, 2025. The copyright holder for this preprint (which was not certified by peer review) is the author/funder, who has granted bioRxiv a license to display the preprint in perpetuity. It is made available under aCC-BY 4.0 International license.

pre_pt_root_id	pref_mn	pref_module	classification_system	putative_NT	ps1	ps2	tp1	tp2	tpn	i1	i2	iii1	iii3	iii4	hg1	hg2	hg3	hg4	b1	b2	b3	MN_syn_total
648518346511116416	iii3	steerA_syn	sensory	unk	10	0	0	0	8	0	0	0	14	0	0	0	0	0	0	0	0	32
648518346477614848	iii3	steerA_syn	sensory	unk	5	0	0	0	3	0	0	0	21	0	0	0	0	0	0	0	0	29
648518346479774848	iii3	steerA_syn	sensory	unk	5	0	0	0	9	0	0	0	14	0	0	0	0	0	0	0	0	28
648518346492104960	iii3	steerA_syn	sensory	unk	4	0	0	0	6	0	0	0	17	0	0	0	0	0	0	0	0	27
648518346481374592	iii3	steerA_syn	sensory	unk	0	0	0	0	10	0	0	0	13	0	0	0	0	0	0	0	0	23
648518346487778560	iii3	steerA_syn	sensory	unk	4	0	0	0	6	0	0	0	12	0	0	0	0	0	0	0	0	22
648518346488285440	iii3	steerA_syn	sensory	unk	0	0	0	0	3	0	0	0	19	0	0	0	0	0	0	0	0	22
648518346490115840	iii3	steerA_syn	sensory	unk	4	0	0	0	3	0	0	0	15	0	0	0	0	0	0	0	0	22
648518346481370496	iii3	steerA_syn	sensory	unk	0	0	0	0	3	0	0	0	12	0	0	0	0	0	0	0	0	15

Figure S3. Premotor neurons from haltere tract innervating tpn motoneuron

pre_pt_root_id	pref_mn	pref_module	cell_type	putative_NT	dlm1	dlm2	dlm3	dlm4	dlm5	dvm1a	dvm1b	dvm1c	dvm2a	dvm2b	dvm3a	dvm3b	ps1	ps2	tp1	tp2	tpn	i1	i2	iii1	iii3	iii4	hg1	hg2	hg3	hg4	b1	b2	b3	MN_syn_total
648518346493194496	DLM_3	DLM_syn	6A	GABA	6	8	13	5	6	0	0	0	0	0	0	0	6	0	10	7	0	0	0	0	0	0	0	0	0	0	0	0	0	61
648518346492652928	DVM_1c	DLM_syn	19B	ACh	21	8	14	29	32	30	5	45	0	0	0	13	0	0	14	8	0	0	0	0	0	0	0	0	0	0	0	0	0	219
648518346495531904	DVM_1c	DLM_syn	19B	ACh	7	7	6	9	11	8	0	17	0	0	0	0	0	0	13	3	0	0	0	0	0	0	0	0	0	0	0	0	0	81
648518346495707008	PS1	tension_syn	uk	unk	0	9	11	16	4	44	7	57	6	0	21	34	155	0	3	13	19	3	0	0	5	0	0	0	0	0	0	0	0	407
648518346489364864	PS1	tension_syn	5B	GABA	0	0	0	0	0	0	0	0	0	0	0	0	191	0	111	0	5	0	0	0	0	0	0	0	0	0	0	8	0	315
648518346509978880	PS1	tension_syn	19B	ACh	0	0	0	0	0	0	0	0	0	0	0	0	103	0	100	19	0	0	0	0	0	0	0	0	0	0	0	0	0	222
648518346512598400	PS1	tension_syn	19B	ACh	0	0	3	0	0	0	0	0	0	0	0	0	139	0	32	16	0	0	0	0	0	8	0	0	0	0	0	0	0	198
648518346513917952	PS1	tension_syn	uk	unk	0	8	16	10	0	30	3	18	3	0	7	12	78	0	0	4	3	0	0	0	0	0	0	0	0	0	0	0	0	192
648518346496711424	PS1	tension_syn	19B	ACh	0	0	0	0	0	0	0	0	0	0	0	0	106	0	9	10	0	0	0	0	0	4	0	0	0	0	0	0	0	129
648518346514394368	PS1	tension_syn	11B	GABA	0	0	0	0	0	0	0	0	0	0	0	0	109	0	6	6	0	0	0	0	0	0	0	0	0	0	0	8	0	129
648518346494550016	PS1	tension_syn	19B	ACh	0	0	0	0	0	0	0	0	0	0	0	0	113	0	3	7	0	4	0	0	0	0	0	0	0	0	0	0	0	127
648518346498208128	PS1	tension_syn	3B	GABA	0	0	0	0	0	0	0	0	0	0	0	0	102	0	0	8	4	0	0	0	11	0	0	0	0	0	0	0	0	125
648518346477886976	PS1	tension_syn	11A	ACh	0	0	0	0	0	0	0	0	0	0	0	0	115	0	3	3	0	0	0	0	0	3	0	0	0	0	0	0	0	124
648518346500910720	PS1	tension_syn	3B	GABA	0	0	0	0	0	0	0	0	0	0	0	0	91	0	20	11	0	0	0	0	0	0	0	0	0	0	0	0	0	122
648518346487435136	PS1	tension_syn	11B	GABA	0	0	0	0	0	0	0	0	0	0	0	0	90	0	17	7	3	0	0	3	0	0	0	0	0	0	0	0	0	120
648518346496690944	PS1	tension_syn	17A	ACh	0	0	0	0	0	0	0	16	0	0	0	0	31	3	0	26	5	0	0	19	0	0	0	0	11	0	0	0	0	111
648518346494056192	PS1	tension_syn	11A	ACh	0	0	0	0	0	0	0	0	0	0	0	0	68	0	0	13	3	0	0	0	21	0	0	0	0	0	0	0	0	105
648518346493991680	PS1	tension_syn	11B	GABA	0	0	0	0	0	0	0	0	0	0	0	0	79	0	10	3	0	0	0	0	0	0	0	0	0	0	0	0	0	92
648518346483546752	PS1	tension_syn	17A	ACh	0	0	0	0	0	0	0	0	0	0	0	0	26	0	0	17	3	0	0	24	0	0	0	0	0	0	0	0	0	70
648518346492480640	PS1	tension_syn	12B	GABA	0	0	0	0	0	0	0	0	0	0	0	0	6	0	3	4	0	0	0	0	0	0	0	6	5	0	0	0	0	24
648518346487572480	dtpmn_u	tension_syn	19B	ACh	0	0	0	0	0	0	0	0	0	0	0	0	72	0	104	12	0	0	0	0	0	0	0	0	0	0	0	0	0	188
648518346526231808	dtpmn_u	tension_syn	11B	GABA	0	0	0	0	0	0	0	0	0	0	0	0	42	0	73	5	0	0	0	0	0	0	0	0	0	0	0	3	0	123
648518346494205056	dtpmn_u	tension_syn	11A	ACh	0	0	0	0	0	0	0	0	0	0	0	0	29	0	77	10	0	0	0	0	0	0	0	0	0	3	0	0	0	119
648518346490917120	dtpmn_u	tension_syn	19B	ACh	0	0	0	0	4	0	0	0	0	0	0	0	26	0	66	5	0	0	0	0	0	0	0	0	0	0	0	0	0	101
648518346465967360	dtpmn_u	DLM_syn	6B	GABA	13	3	12	11	14	0	0	0	0	0	0	0	0	0	41	0	3	0	0	0	0	0	0	0	0	0	3	0	0	100
648518346493225472	dtpmn_u	tension_syn	11B	GABA	0	0	0	0	3	0	0	0	0	0	0	0	32	0	55	7	0	0	0	0	0	0	0	0	0	0	0	0	0	97
648518346501803904	dtpmn_u	tension_syn	19B	ACh	0	0	0	0	0	0	0	3	0	0	0	0	0	17	46	28	0	0	0	0	0	0	0	0	0	0	0	0	0	94
648518346496520320	dtpmn_u	tension_syn	19B	ACh	0	0	0	0	0	0	0	0	0	0	0	0	9	12	51	11	0	0	0	0	0	0	0	0	5	0	0	0	0	88
648518346518340480	dtpmn_u	tension_syn	8A	Glu	0	0	0	0	0	0	0	0	0	0	0	0	0	0	36	27	0	0	0	0	0	0	0	0	6	0	0	0	0	69
648518346479754496	dtpmn_u	tension_syn	19B	ACh	0	0	0	0	0	0	0	0	0	0	0	0	0	13	42	12	0	0	0	0	0	0	0	0	0	0	0	0	0	67
648518346472977536	dtpmn_u	tension_syn	6A	GABA	5	0	6	0	3	0	0	4	0	0	4	0	6	0	15	9	0	0	0	0	0	0	0	0	0	0	0	0	0	52
648518346507298688	dtpmn_u	tension_syn	2A	Glu	0	0	0	0	0	0	0	0	0	0	0	0	0	3	30	6	0	0	0	0	0	0	0	0	0	0	0	0	0	39
648518346498502656	dtpmn_u	tension_syn	8A	Glu	0	0	0	0	0	0	0	0	0	0	0	0	0	0	23	13	0	0	0	0	0	0	0	0	0	0	0	0	0	36
648518346506616064	dtpmn_u	tension_syn	8A	Glu	0	0	0	0	0	0	0	0	0	0	0	0	7	0	21	6	0	0	0	0	0	0	0	0	0	0	0	0	0	34
648518346497999744	dtpmn_u	tension_syn	8A	Glu	0	0	0	0	0	0	0	0	0	0	0	0	0	0	19	11	0	0	0	0	0	0	0	0	0	0	0	0	0	30
648518346485424640	dtpmn_u	tension_syn	8A	Glu	0	0	0	0	0	0	0	0	0	0	0	0	0	0	19	6	0	0	0	0	0	0	0	0	0	0	0	0	0	25
648518346488672512	dtpmn_u	tension_syn	19B	ACh	0	0	0	0	0	0	0	0	0	0	0	0	0	0	14	5	0	0	0	0	0	0	0	0	0	0	0	0	0	19
648518346486472320	tp2	tension_syn	11A	ACh	0	0	0	0	0	9	0	11	0	0	21	14	0	0	4	72	0	0	0	0	0	0	0	0	0	0	0	0	11	142
648518346501086464	tp2	tension_syn	17A	ACh	0	0	0	0	0	0	0	0	0	0	0	0	0	5	23	28	0	0	0	0	0	0	0	0	0	0	0	18	0	74
648518346492152832	tp2	tension_syn	8A	Glu	0	0	0	0	0	0	0	0	0	0	0	0	0	6	25	39	0	0	0	0	0	0	0	0	0	0	0	0	0	70
648518346502422144	tp2	tension_syn	7B	ACh	0	0	0	0	0	0	0	0	0	0	0	0	0	0	15	28	8	0	0	0	19	0	0	0	0	0	0	0	0	70
648518346494030592	tp2	tension_syn	11B	GABA	0	0	0	0	0	0	0	3	0	0	0	0	0	0	0	39	10	0	0	0	0	0	0	0	0	0	0	0	0	52
648518346500883840	tp2	tension_syn	11B	GABA	0	0	0	0	0	0	0	0	0	0	0	0	0	0	4	39	7	0	0	0	0	0	0	0	0	0	0	0	0	50
648518346495524480	tp2	tension_syn	17A	ACh	0	0	0	0	0	6	0	3	0	0	0	0	0	0	8	10	0	0	0	0	0	0	0	0	0	0	0	5	0	32
648518346512196352	tp2	tension_syn	uk	unk	0	0	0	0	0	5	0	0	0	0	0	0	0	0	5	20	0	0	0	0	0	0	0	0	0	0	0	0	0	30
648518346515177088	tp2	tension_syn	11B	GABA	0	0	0	0	0	0	0	0	0	0	0	0	0	0	4	12	3	0	0	0	0	0	0	0	4	0	0	0	0	23
648518346478847744	tp2	tension_syn	11B	GABA	0	0	0	0	0	0	0	0	0	0	0	0	0	3	3	10	0	0	0	0	0	0	0	0	3	0	0	0	0	19
648518346496080896	tpn	steerA_syn	11B	GABA	0	0	0	0	0	0	0	0	0	0	0	0	0	0	0	7	54	0	0	20	15	0	0	0	0	3	0	0	0	99
648518346480111488	tpn	steerA_syn	11B	GABA	0	0	0	0	0	0	0	0	0	0	0	0	0	0	0	5	44	0	0	30	5	0	0	0	0	3	0	0	0	87
648518346500015360	tpn	steerA_syn	11A	ACh	0	0	0	0	0	0	0	0	0	0	0	0	0	0	0	9	19	0	0	0	4	0	0	0	0	0	0	0	0	32
648518346479239040	i2_u	tension_syn	11B	GABA	0	0	0	0	0	4	7	3	0	0	6	8	0	8	0	23	5	0	31	0	0	0	0	0	6	9	0	0	0	110
648518346507590272	iii3	steerA_syn	7B	ACh	0	0	0	0	0	0	0	0	0	0	0	0	0	0	36	24	36	0	0	0	59	0	0	0	0	0	0	0	0	155
648518346511910784	iii3	steerA_syn	7B	ACh	0	0	0	0	0	0	0	0	0	0	0	0	0	0	25	12	50	0	0	0	62	0	0	0	0	0	0	0	0	149
648518346510991232	iii3	steerA_syn	8A	Glu	0	0	0	0	0	0	0	0	0	0	0	0	43	0	0	8	15	0	0	16	48	0	0	0	0	0	0	0	0	130
648518346474542336	iii3	steerA_syn	8A	Glu	0	0	0	0	0	0	0	0	0	0	0	0	30	0	0	6	16	0	0	5	41	0	0	0	0	0	0	0	0	98
648518346493144832	iii3	steerA_syn	6A	GABA	0	0	0	0	0	0	0	0	0	0	0	0	0	0	0	5	20	0	0	3	41	0	0	0	0	0	0	0	0	69
648518346492648064	iii3	steerA_syn	7B	ACh	0	0	0	0	0	0	0	0	0	0	0	0	0	0	14	0	20	0	0	0	27	0	0	0	0	0	0	0	0	61
648518346479820032	hg3	steerD_syn	17A	ACh	0	0	0	0	0	0	0	0	0	0	0	0	6	0	5	4	0	0	0	0	0	10	15	0	59	0	0	47	0	146
648518346478123264	hg3	steerD_syn	7B	ACh	0	0	0	0	0	0	0	4	0	0	0	0	0	0	13	3	0	4	44	0	0	0	0	8	54	6	0	0	0	136

Figure S4. Local premotor neurons innervating tp motoneurons

bioRxiv preprint doi: https://doi.org/10.1101/2025.11.02.686144; this version posted November 8, 2025. The copyright holder for this preprint (which was not certified by peer review) is the author/funder, who has granted bioRxiv a license to display the preprint in perpetuity. It is made available under aCC-BY 4.0 International license.

Supplementary Note 2: Maximum intensity projection of split driver lines

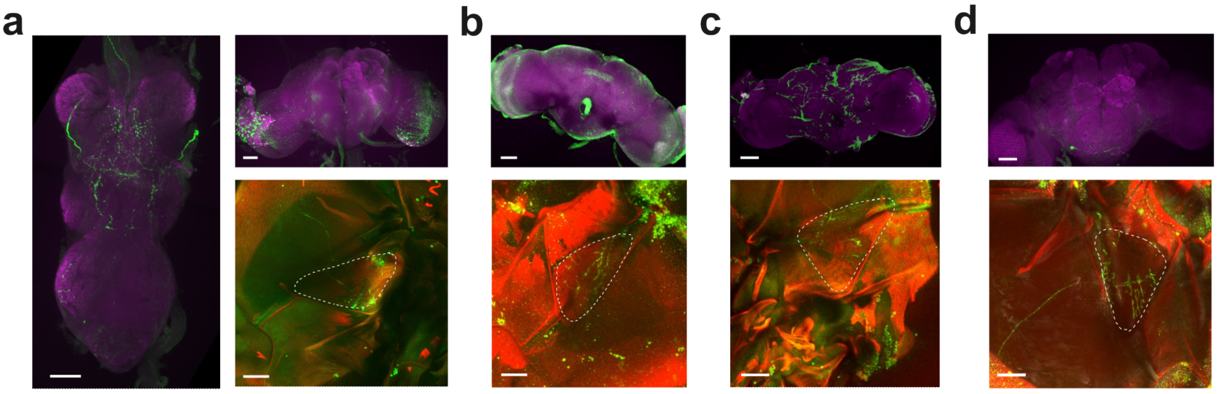

Figure S5. Maximum intensity projection (MIP) images of the split driver lines tp1,tp1-SG,tp2 and tpN (a) Left - A Maximum intensity projection (MIP) VNC image from a tp1-SG > CsChrimson fly. Green corresponds to mVenus, purple to DNCad (neuropil). Top right- MIP brain image from a tp1-SG > CsChrimson fly. Bottom right - Phalloidin-stained thoracic hemisection from a tp1-SG > CsChrimson fly showing wing musculature, with both phalloidin (red) and GFP (green) expression innervating the tp1 muscle (outlined with white dashed lines). (b) Top - same as (a) top right but with tp1-GAL4 > CsChrimson. Bottom - same as (a) bottom left except with tp1-GAL4 > CsChrimson. (c) Top - same as (a) top right but with tp2-GAL4 > CsChrimson. Bottom - same as (a) bottom left except with tp2-GAL4 > CsChrimson. (d) Top - same as (a) top right but with tpN-GAL4 > CsChrimson. Bottom - same as (a) bottom left except with tpN-GAL4 > CsChrimson. All scale bars 50 μm. See Table 1 for full fly genotypes

bioRxiv preprint doi: https://doi.org/10.1101/2025.11.02.686144; this version posted November 8, 2025. The copyright holder for this preprint (which was not certified by peer review) is the author/funder, who has granted bioRxiv a license to display the preprint in perpetuity. It is made available under aCC-BY 4.0 International license.

880

Supplementary Note 3: Lift Maximization Model Predictions

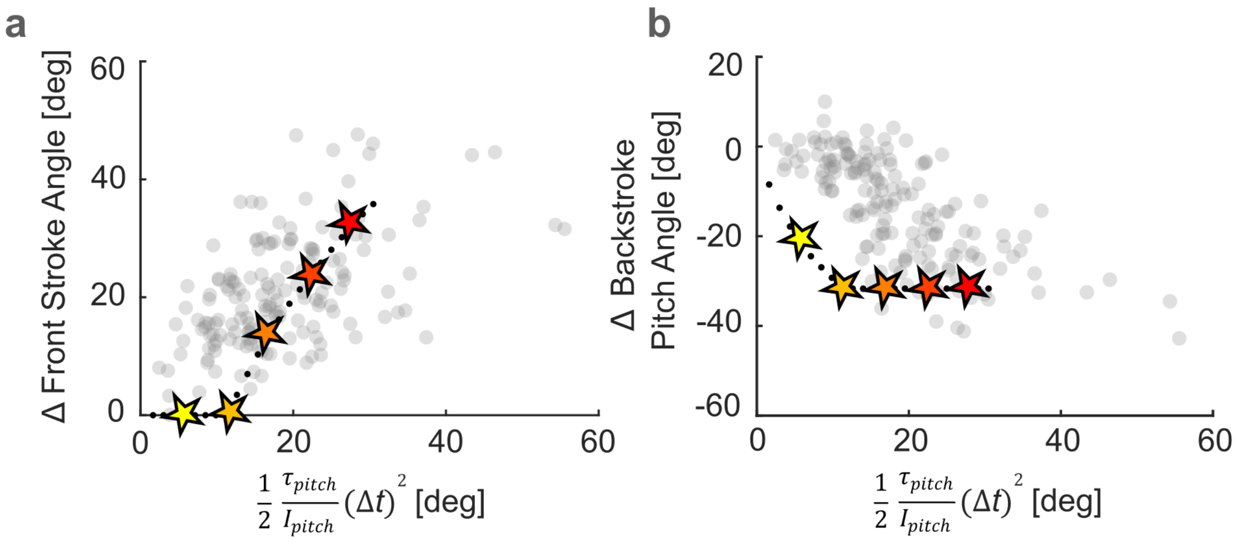

Figure S6. Lift maximization model prediction (a) The backstroke wing pitch response and (b) front stroke response generated from the lift maximization model in Fig. 2 (n). The grey dots are experimental data. The dotted lines represent the model prediction, and the star symbols correspond to the example contours discussed in the main text.

bioRxiv preprint doi: https://doi.org/10.1101/2025.11.02.686144; this version posted November 8, 2025. The copyright holder for this preprint (which was not certified by peer review) is the author/funder, who has granted bioRxiv a license to display the preprint in perpetuity. It is made available under aCC-BY 4.0 International license.

Supplementary Note 4: Tp muscles optogenetic activation and inhibition analysis

Table 2. Statistical results for Fig. 3. Summary of nonparametric group comparisons for wing kinematics and behavioral responses under tp1 inhibition and activation. H refers to the Kruskal–Wallis test statistic; p is the corresponding uncorrected significance value. η^2 indicates the effect size estimate for Kruskal–Wallis tests, and h denotes Cohen's h for pairwise proportion comparisons. Post hoc p-values are from multiple comparisons test with Dunnett correction (for panels a–d, f–i), or Bonferroni-corrected pairwise proportion tests (for panels e, j). See Supplementary Table 3 for effect size interpretations.

Panel	Measure	Comparison	H	p	η^2 / h	Post hoc p
(a, b)	Wing pitch (Small perturbation)	control vs tp1<br>control vs tp1-alt	0.97	0.616	0	0.991<br>0.676
	Wing stroke (Small perturbation)	control vs tp1<br>control vs tp1-alt	4.76	0.093	0.084	0.999<br>0.132
(c, d)	Wing pitch (Big perturbation)	control vs tp1<br>control vs tp1-alt	9.58	0.008	0.100	0.026<br>0.008
	Wing stroke (Big perturbation)	control vs tp1<br>control vs tp1-alt	4.05	0.132	0.027	0.261<br>0.094
(e)	Correction (>50%)	control vs tp1<br>control vs tp1-alt	—	0.0075<br><0.001	$h = -0.755$<br>$h = -1.446$	—<br>—
(f, g)	Wing pitch (Small perturbation)	control vs tp1<br>control vs tp1-alt	9.41	0.009	0.151	0.008<br>0.023
	Wing stroke (Small perturbation)	control vs tp1<br>control vs tp1-alt	9.50	0.009	0.153	0.101<br>0.004
(h, i)	Wing pitch (Big perturbation)	control vs tp1<br>control vs tp1-alt	0.12	0.944	0	0.926<br>0.951
	Wing stroke (Big perturbation)	control vs tp1<br>control vs tp1-alt	2.71	0.258	0.012	0.958<br>0.223
(j)	Over-correction (>10%)	control vs tp1<br>control vs tp1-alt	—	0.0030<br>0.0200	$h = 1.160$<br>$h = 0.890$	—<br>—

Table 3. Interpretation of Cohen's h effect sizes used in Fig. 3

Panel	Comparison	Cohen's h	Interpretation
(e)	control vs tp1	-0.755	Large
	control vs tp1-alt	-1.446	Very large
(j)	control vs tp1	1.160	Very large
	control vs tp1-alt	0.890	Large

bioRxiv preprint doi: https://doi.org/10.1101/2025.11.02.686144; this version posted November 8, 2025. The copyright holder for this preprint (which was not certified by peer review) is the author/funder, who has granted bioRxiv a license to display the preprint in perpetuity. It is made available under aCC-BY 4.0 International license.

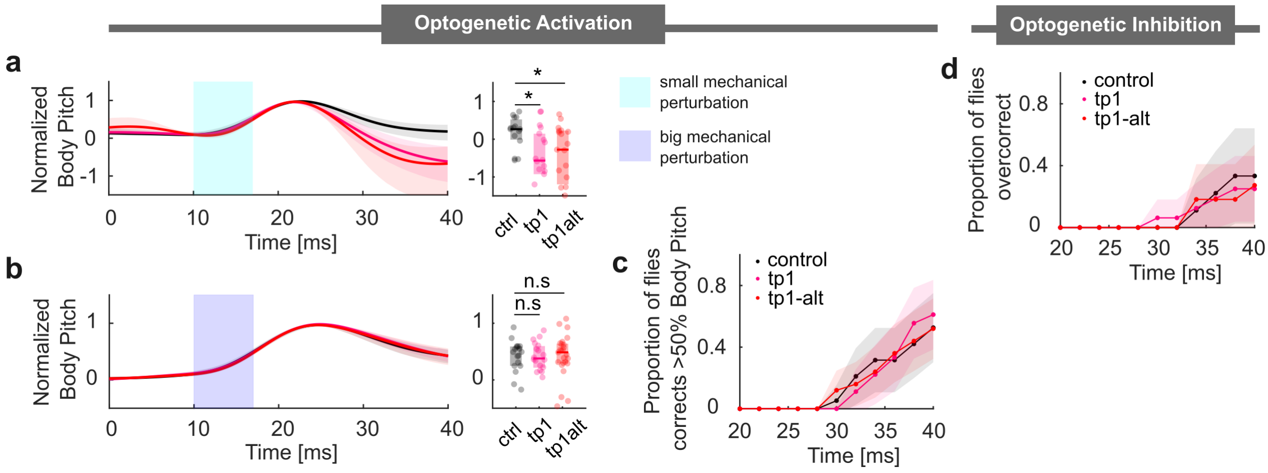

Figure S7. Additional tp1 activation and inhibition plots (a and b) Left: The normalized body pitch angle of control and tp1-activated flies along the pitch axis during a correction maneuver for small and big pitch perturbation. Right: Normalized body pitch angle at time 40ms. (c) The proportion of flies correcting more than 50% of control flies body deflection amplitude at time 40ms. (d) The proportion of flies over-correcting more than 10% of control flies body deflection amplitude at time 40ms. Statistical significance for (a)-(b) is determined via the Kruskal-Wallis test with Dunn's post hoc multiple comparisons. Statistical significance for (c) and (d) is determined via the Proportional test with Bonferroni correction(***, $p < .001$; **, $p < .01$; *, $p < .05$). Optogenetic inhibition small perturbation: control n=12, tp1 n= 24, tp1-alt n=15. Optogenetic activation small perturbation: control, n=16, tp1 n= 17, tp1-alt n= 11. Optogenetic activation big perturbation: control, n=19, tp1 n= 16, tp1-alt n= 11).

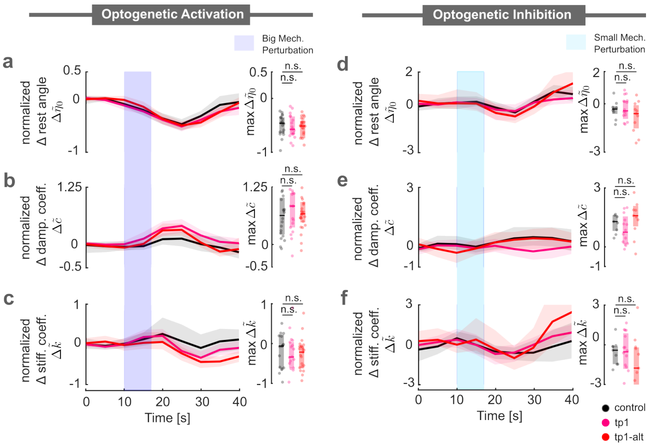

Figure S8. Additional tp1 torsional spring fit plots (a) Left: Normalized change in the torsional spring rest angle: Comparison of control and tp1-activated flies in response to large pitch perturbations. Right: Maximum change in normalized torsional spring rest angle. (b) Left: Normalized change in the torsional spring damping coefficient: Comparison of control and tp1-activated flies in response to large pitch perturbations. Right: Maximum change in normalized torsional spring damping coefficient. (c) Left: Normalized change in the torsional spring stiffness constant: Comparison of control and tp1-activated flies in response to large pitch perturbations. Right: Maximum change in normalized torsional spring stiffness constant. (d) Left: Normalized change in the torsional spring rest angle: Comparison of control and tp1-inhibited flies in response to small pitch perturbations. Right: Maximum change in normalized torsional spring rest angle. (e) Left: Normalized change in the torsional spring damping coefficient: Comparison of control and tp1-inhibited flies in response to small pitch perturbations. Right: Maximum change in normalized torsional spring damping coefficient. (f) Left: Normalized change in the torsional spring stiffness constant: Comparison of control and tp1-inhibited flies in response to small pitch perturbations. Right: Maximum change in normalized torsional spring stiffness constant. Statistical significance for (a)-(f) is determined via the Kruskal-Wallis test with Dunn's post hoc multiple comparisons. (***, $p < .001$; **, $p < .01$; *, $p < .05$). Optogenetic activation big perturbation: control, n=19, tp1 n= 16, tp1-alt n= 11. Optogenetic inhibition small perturbation: control n=12, tp1 n= 24, tp1-alt n= 15.

bioRxiv preprint doi: https://doi.org/10.1101/2025.11.02.686144; this version posted November 8, 2025. The copyright holder for this preprint (which was not certified by peer review) is the author/funder, who has granted bioRxiv a license to display the preprint in perpetuity. It is made available under aCC-BY 4.0 International license.

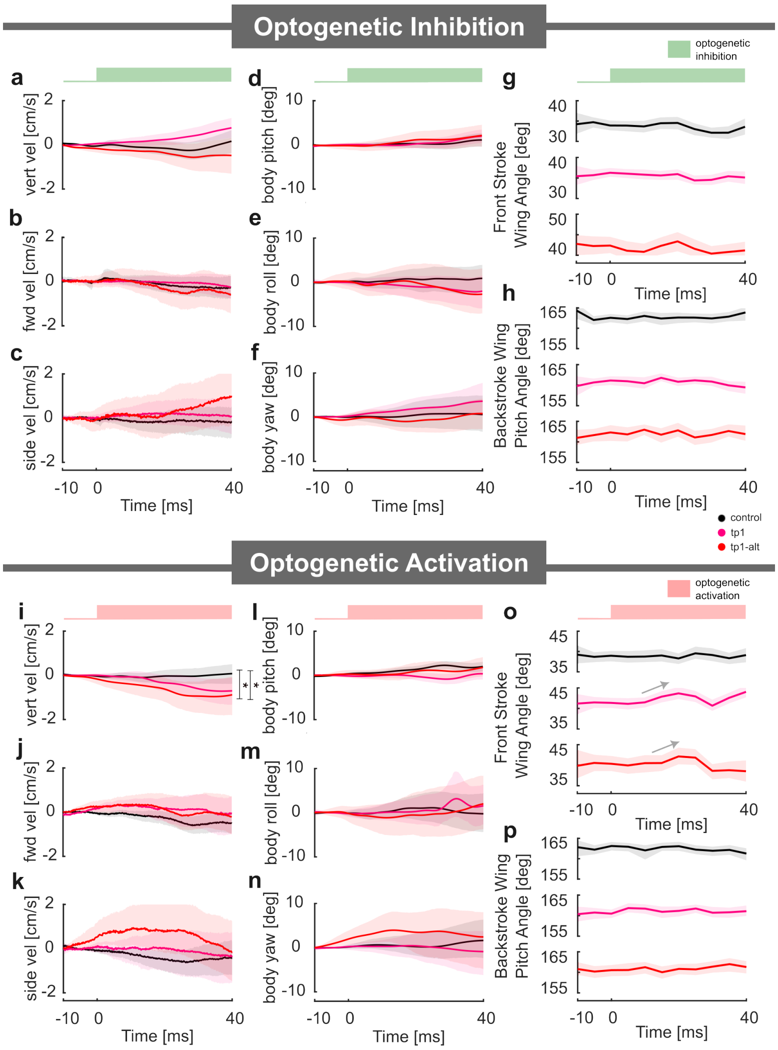

Figure S9. tp1 inhibition and activation data. (First column: a, b, c, i, j and k) Translational velocities (Vertical, forward, and side) of control and tp1-inhibited/activated flies. (Second column: d, e, f, l, m and n) Angular orientation (body pitch, body roll, and body yaw) of control and tp1-inhibited/activated flies. (Third column: g, h, o, and p) Front stroke wing angle and backstroke pitch angle of control and tp1-inhibited/activated flies. In (i) tp1- activated flies show a downward drift compared to controls. A linear slope was estimated for each time series within the groups to quantify this change. Distributionally, the slope for tp1-activated flies exhibits a heavier tail. (Proportion test at the 10% percentile of control, $p < .01$). The observed vertical velocity drift in tp1-activated flies can be attributed to a slight decrease in the front stroke wing angle (panel o), which leads to reduced lift generation. Sample sizes for optogenetic inhibition: control n= 94, tp1 n= 93 and tp1-alt n= 46. Sample sizes for optogenetic activation: control, n=93, tp1 n= 93 and tp1-alt n= 57

bioRxiv preprint doi: https://doi.org/10.1101/2025.11.02.686144; this version posted November 8, 2025. The copyright holder for this preprint (which was not certified by peer review) is the author/funder, who has granted bioRxiv a license to display the preprint in perpetuity. It is made available under aCC-BY 4.0 International license.

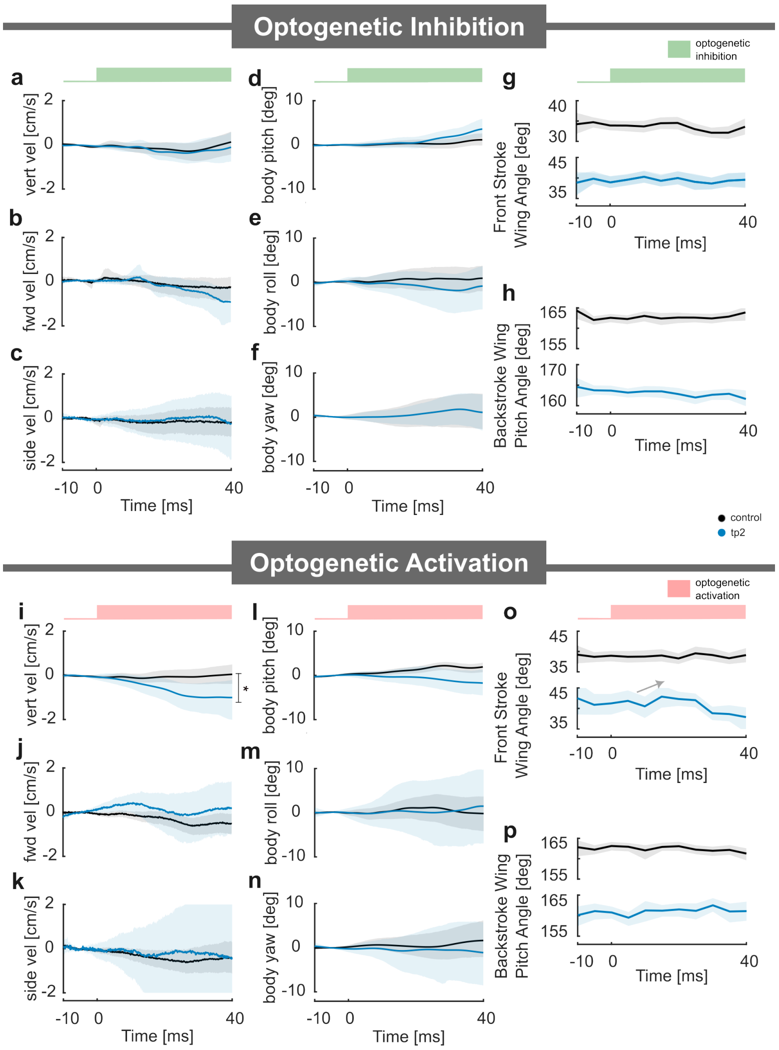

Figure S10. tp2 free flight inhibition and activation data. (First column: a, b, c, i, j and k) Translational velocities (Vertical, forward, and side) of control and tp2-inhibited/activated flies. (Second column: d, e, f, l, m and n) Angular orientation (body pitch, body roll, and body yaw) of control and tp2-inhibited/activated flies. (Third column: g, h, o, and p) Front stroke wing angle and backstroke pitch angle of control and tp1-inhibited/activated flies. In (i) tp2- activated flies show a downward drift compared to controls. A linear slope was estimated for each time series within the groups to quantify this change. Distributionally, the slope for tp2-activated flies exhibits a heavier tail. (Proportion test at the 10% percentile of control, $p < .01$). The observed vertical velocity drift in tp1-activated flies can be attributed to a slight decrease in the front stroke wing angle (panel o), which leads to reduced lift generation Sample sizes for optogenetic inhibition: control n= 94, tp2 n= 59. Sample sizes for optogenetic activation: control, n=93, tp2 n= 39

bioRxiv preprint doi: https://doi.org/10.1101/2025.11.02.686144; this version posted November 8, 2025. The copyright holder for this preprint (which was not certified by peer review) is the author/funder, who has granted bioRxiv a license to display the preprint in perpetuity. It is made available under aCC-BY 4.0 International license.

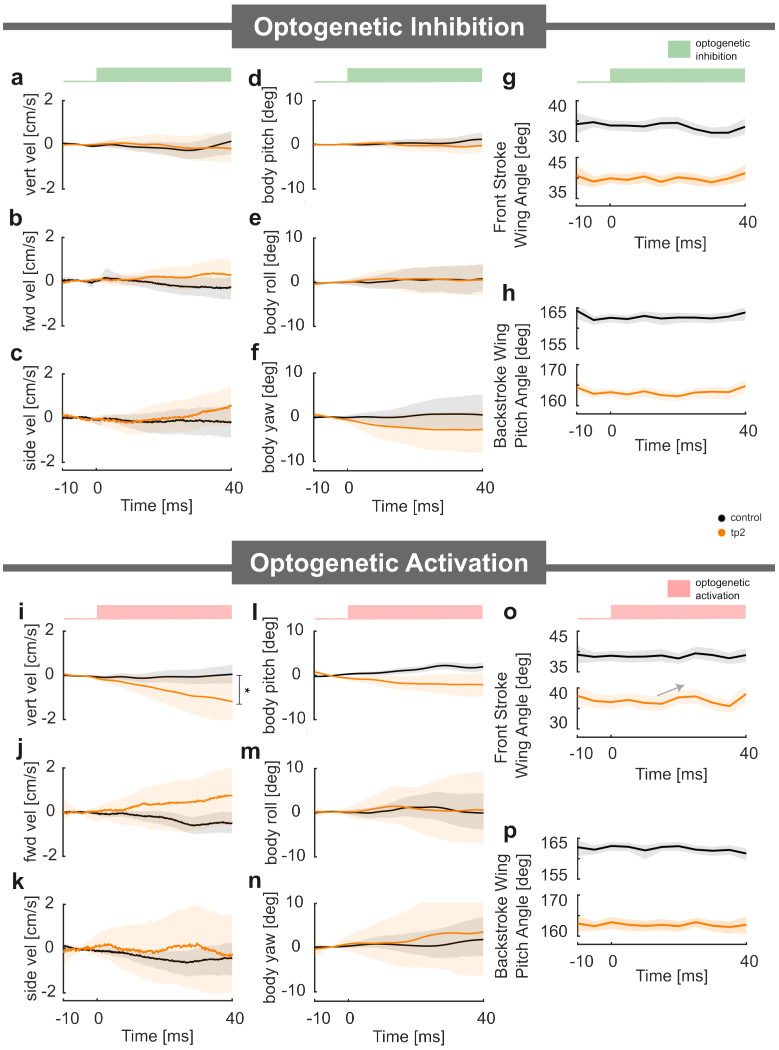

Figure S11. tpN free flight inhibition and activation data. (First column: a, b, c, i, j and k) Translational velocities (Vertical, forward, and side) of control and tpN-inhibited/activated flies. (Second column: d, e, f, l, m and n) Angular orientation (body pitch, body roll, and body yaw) of control and tpN-inhibited/activated flies. (Third column: g, h, o, and p) Front stroke wing angle and backstroke pitch angle of control and tpN-inhibited/activated flies. In (i) tpN- activated flies show a downward drift compared to controls. A linear slope was estimated for each time series within the groups to quantify this change. Distributionally, the slope for tpN-activated flies exhibits a heavier tail. (Proportion test at the 10% percentile of control, $p <.01$). The observed vertical velocity drift in tpN-activated flies can be attributed to a slight decrease in the front stroke wing angle (panel o), which leads to reduced lift generation Sample sizes for optogenetic inhibition: control n= 94, tpN n= 54. Sample sizes for optogenetic activation: control, n=93, tpN n= 27

bioRxiv preprint doi: https://doi.org/10.1101/2025.11.02.686144; this version posted November 8, 2025. The copyright holder for this preprint (which was not certified by peer review) is the author/funder, who has granted bioRxiv a license to display the preprint in perpetuity. It is made available under aCC-BY 4.0 International license.

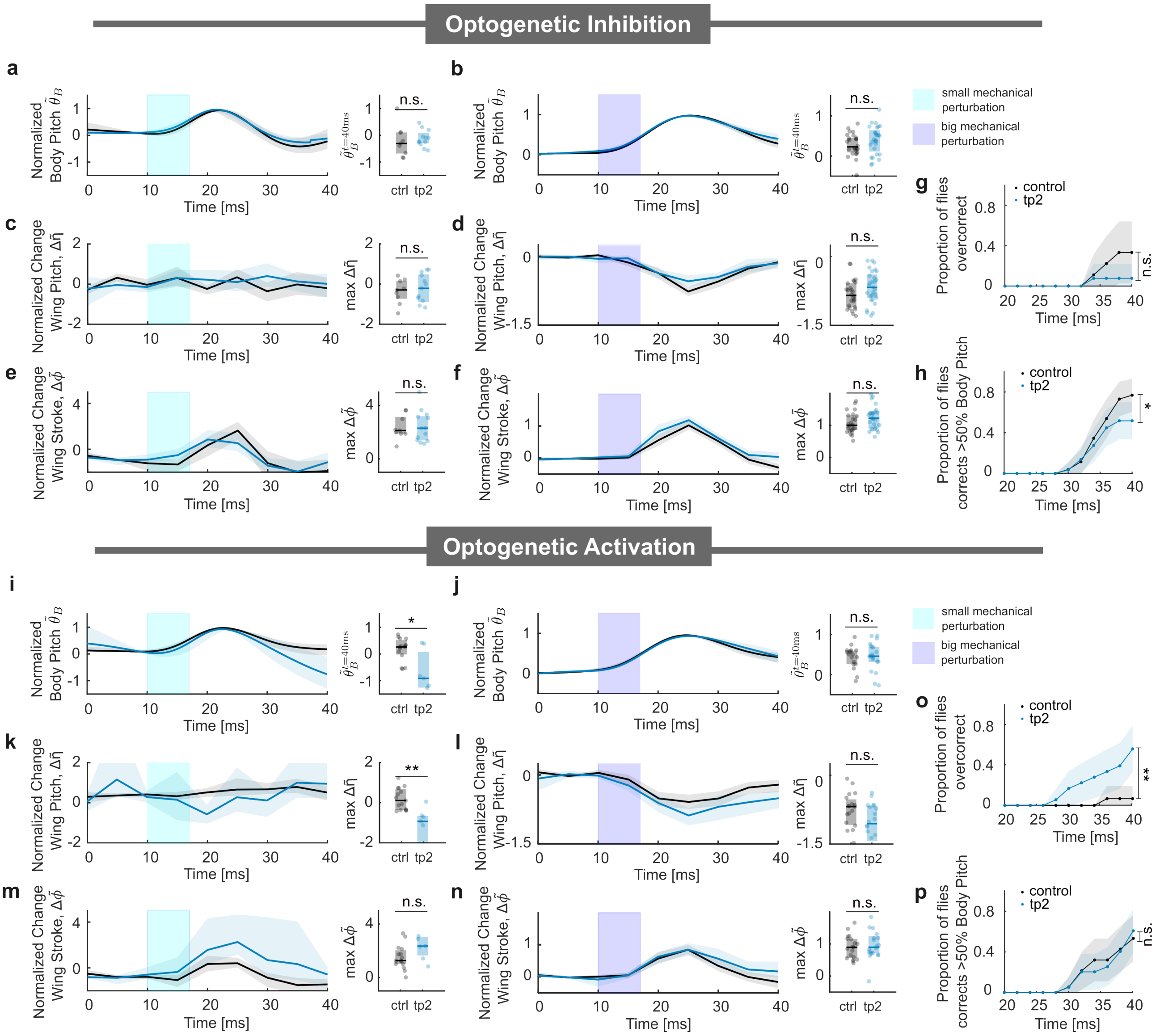

Figure S12. tp2 inhibition and activation data. (a and b) Left: Normalized body pitch angle of control and tp2-inhibited flies along the pitch axis during a correction maneuver for small and big pitch perturbations. Right: Normalized body pitch angle at time 40ms. (c and d) Left: Normalized change in backstroke wing pitch response: Comparison of control and tp2-inhibited flies in response to small and big pitch perturbations. Right: Maximum change in normalized wing pitch response. (e and f) Left: Normalized change in front stroke response: Comparison of control and tp2-inhibited flies in response to small and big pitch perturbations. Right: Maximum change in normalized wing stroke response (g) The proportion of flies over-correcting more than 10% of control flies body deflection amplitude at time 40ms. (h) The proportion of flies that correct more than 50 % body deflection amplitude during a big pitch perturbation as a function of time. (i and j) Left: Normalized body pitch angle of control and tp2-activated flies along the pitch axis during a correction maneuver for small and big pitch perturbations. Right: Normalized body pitch angle at time 40ms. (k and l) Normalized change in backstroke wing pitch response: Comparison of control and tp2-activated flies in response to small and big pitch perturbations. Right: Maximum change in normalized wing pitch response. (m and n) Left: Normalized change in front stroke response: Comparison of control and tp2-activated flies in response to small and large pitch perturbations. Right: Maximum change in normalized wing stroke response. (o) The proportion of flies over-correcting more than 10% of control flies body deflection amplitude at time 40ms. (p) The proportion of flies that correct more than 50 % body deflection amplitude during a big pitch perturbation as a function of time. Optogenetic inhibition small perturbation: control n=9, tp2 n= 13. Optogenetic inhibition big perturbation: control n=26, tp2 n= 29. Statistical significance for (a)-(f) and (i)-(n) are determined via the Kruskal-Wallis test with Dunn's post hoc multiple comparisons. Statistical significance for (g), (h), (o), and (p) are determined via the Proportional test with Bonferroni correction(***, $p<.001$; **, $p<.01$; *, $p<.05$). Optogenetic activation small perturbation: control, n=15, tp2 n= 7. Optogenetic activation big perturbation: control, n=19, tp2 n= 20

bioRxiv preprint doi: https://doi.org/10.1101/2025.11.02.686144; this version posted November 8, 2025. The copyright holder for this preprint (which was not certified by peer review) is the author/funder, who has granted bioRxiv a license to display the preprint in perpetuity. It is made available under aCC-BY 4.0 International license.

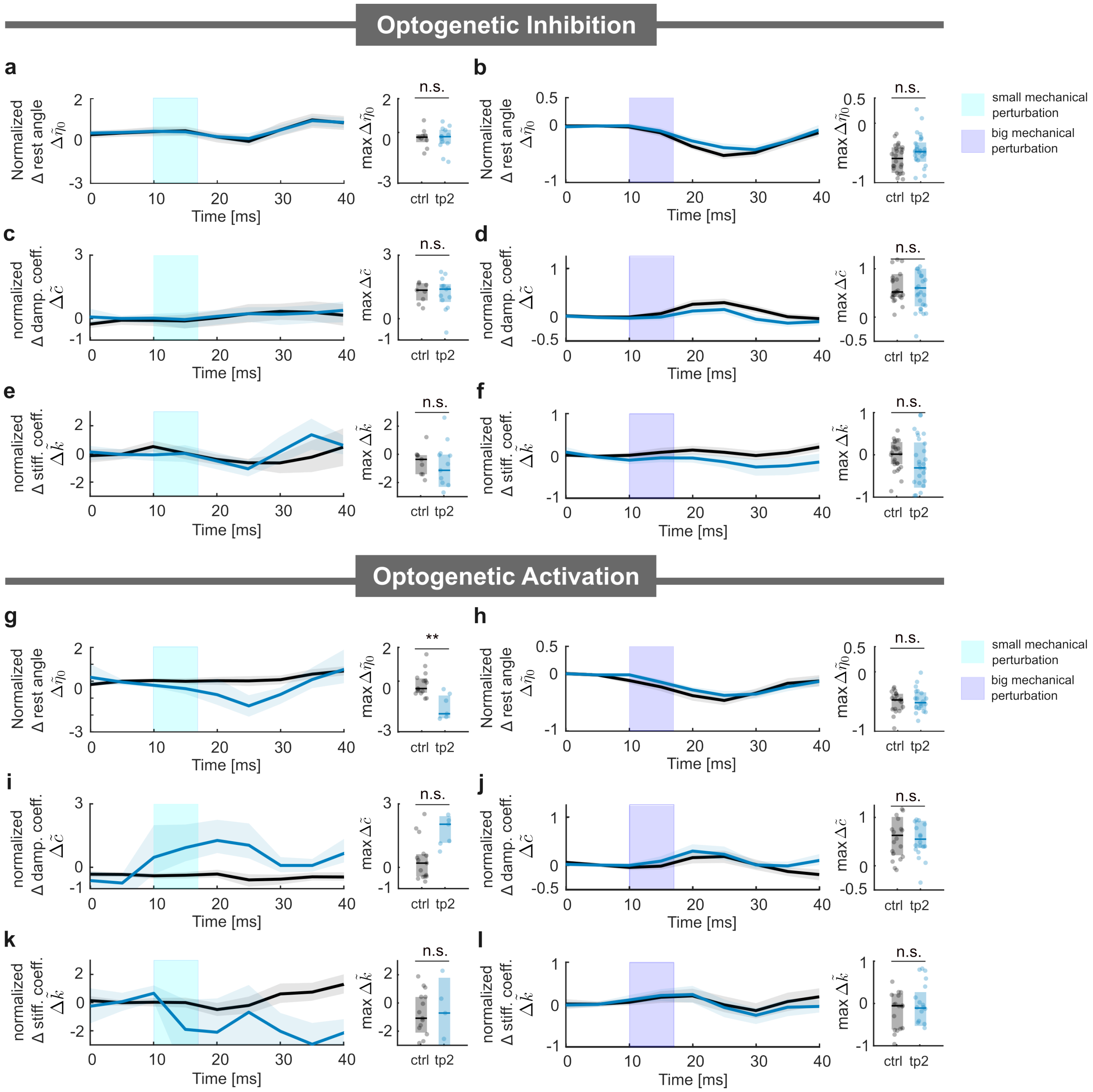

Figure S13. tp2 torsional spring model fit (a,b) Left: Normalized change in the torsional spring rest angle: Comparison of control and tp2-inhibited flies in response to small and large pitch perturbations. Right: Maximum change in normalized torsional spring rest angle. (c,d) Left: Normalized change in the torsional spring damping factor: Comparison of control and tp2-inhibited flies in response to small and large pitch perturbations. Right: Maximum change in normalized torsional spring damping factor. (e,f) Left: Normalized change in the torsional spring stiffness constant: Comparison of control and tp2-inhibited flies in response to small and large pitch perturbations. Right: Maximum change in normalized torsional spring stiffness constant. (g,h) Left: Normalized change in the torsional spring rest angle: Comparison of control and tp2-activated flies in response to small and large pitch perturbations. Right: Maximum change in normalized torsional spring rest angle. (i,j) Left: Normalized change in the torsional spring damping factor: Comparison of control and tp2-activated flies in response to small and large pitch perturbations. Right: Maximum change in normalized torsional spring damping factor. (k,l) Left: Normalized change in the torsional spring stiffness constant: Comparison of control and tp2-activated flies in response to small and large pitch perturbations. Right: Maximum change in normalized torsional spring stiffness constant. Statistical significance for (a)-(l) is determined via the Kruskal-Wallis test with Dunn's post hoc multiple comparisons. (***, $p < .001$; **, $p < .01$; *, $p < .05$). In (g) we observed a significant change in rest angle relative of tp2 activated flies to control (Kruskal-Wallis test, $p < .001$). In panels (i) and (k), the damping factor and stiffness constant estimations exhibit high variability, even before mechanical perturbation, likely due to the small sample size. Optogenetic inhibition small perturbation: control n=9, tp2 n= 13. Optogenetic inhibition big perturbation: control n=26, tp2 n= 29. Optogenetic activation small perturbation: control, n=15, tp2 n= 6. Optogenetic activation big perturbation: control, n=19, tp2 n= 19

bioRxiv preprint doi: https://doi.org/10.1101/2025.11.02.686144; this version posted November 8, 2025. The copyright holder for this preprint (which was not certified by peer review) is the author/funder, who has granted bioRxiv a license to display the preprint in perpetuity. It is made available under aCC-BY 4.0 International license.

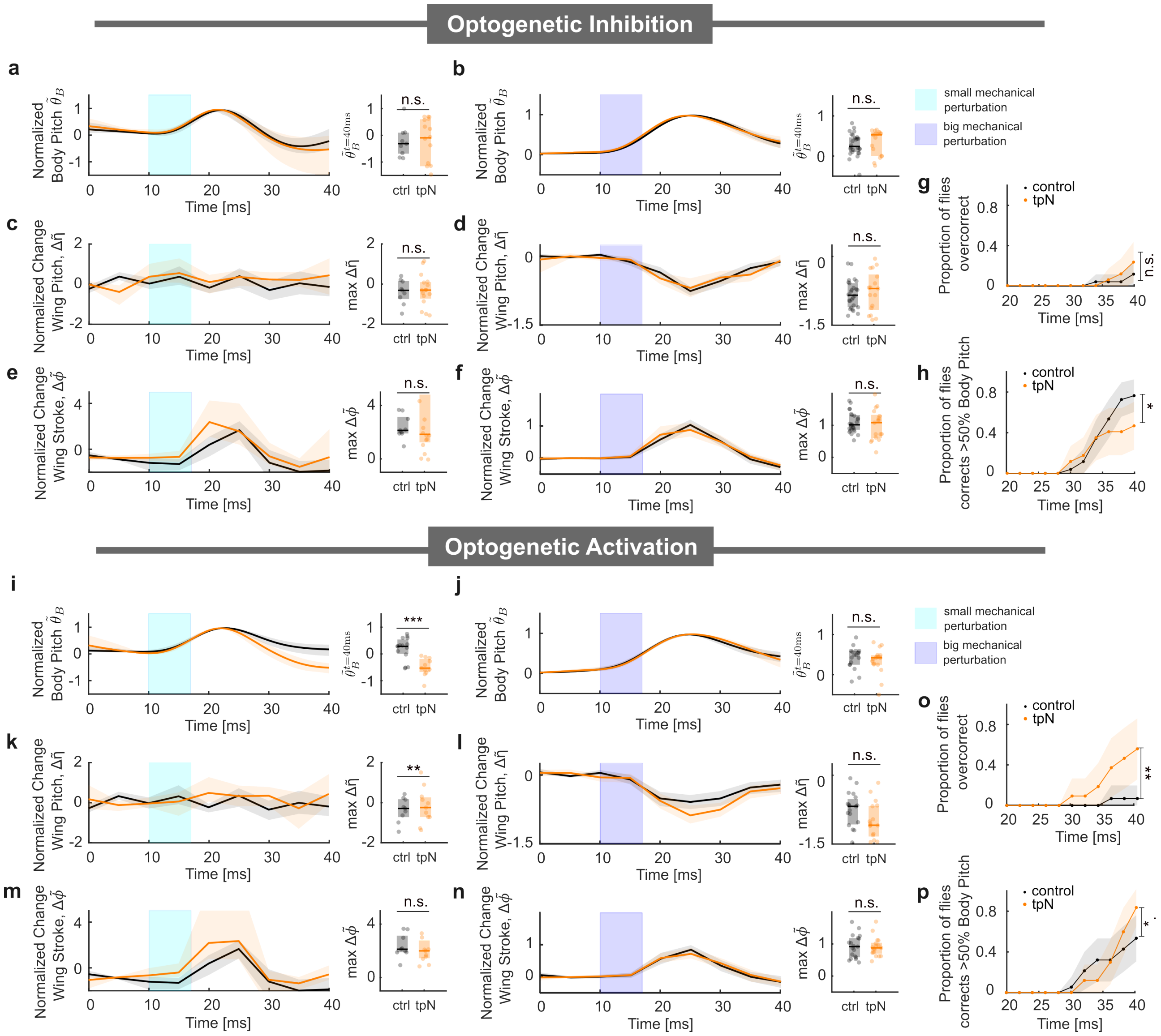

Figure S14. tpN inhibition and activation data. (a and b) Left: Normalized body pitch angle of control and tpN-inhibited flies along the pitch axis during a correction maneuver for small and big pitch perturbations. Right: Normalized body pitch angle at time 40ms. (c and d) Left: Normalized change in backstroke wing pitch response: Comparison of control and tpN-inhibited flies in response to small and big pitch perturbations. Right: Maximum change in normalized wing pitch response. (e and f) Left: Normalized change in front stroke response: Comparison of control and tpN-inhibited flies in response to small and big pitch perturbations. Right: Maximum change in normalized wing stroke response (g) The proportion of flies over-correcting more than 10% of control flies body deflection amplitude at time 40ms. (h) The proportion of flies that correct more than 50 % body deflection amplitude during a big pitch perturbation as a function of time. (i and j) Left: Normalized body pitch angle of control and tpN-activated flies along the pitch axis during a correction maneuver for small and big pitch perturbations. Right: Normalized body pitch angle at time 40ms. (k and l) Normalized change in backstroke wing pitch response: Comparison of control and tpN-activated flies in response to small and big pitch perturbations. Right: Maximum change in normalized wing pitch response. (m and n) Left: Normalized change in front stroke response: Comparison of control and tpN-activated flies in response to small and large pitch perturbations. Right: Maximum change in normalized wing stroke response. (o) The proportion of flies over-correcting more than 10% of control flies body deflection amplitude at time 40ms. (p) The proportion of flies that correct more than 50 % body deflection amplitude during a big pitch perturbation as a function of time. Statistical significance for (a)-(f) and (i)-(n) are determined via the Kruskal-Wallis test with Dunn's post hoc multiple comparisons. Statistical significance for (g), (h), (o), and (p) are determined via the Proportional test with Bonferroni correction(***, $p < .001$; **, $p < .01$; *, $p < .05$). Optogenetic inhibition small perturbation: control n=9, tpN n= 16. Optogenetic inhibition big perturbation: control n=26, tpN n= 17. Optogenetic activation small perturbation: control, n=15, tpN n= 11. Optogenetic activation big perturbation: control, n=19, tpN n= 17

bioRxiv preprint doi: https://doi.org/10.1101/2025.11.02.686144; this version posted November 8, 2025. The copyright holder for this preprint (which was not certified by peer review) is the author/funder, who has granted bioRxiv a license to display the preprint in perpetuity. It is made available under aCC-BY 4.0 International license.

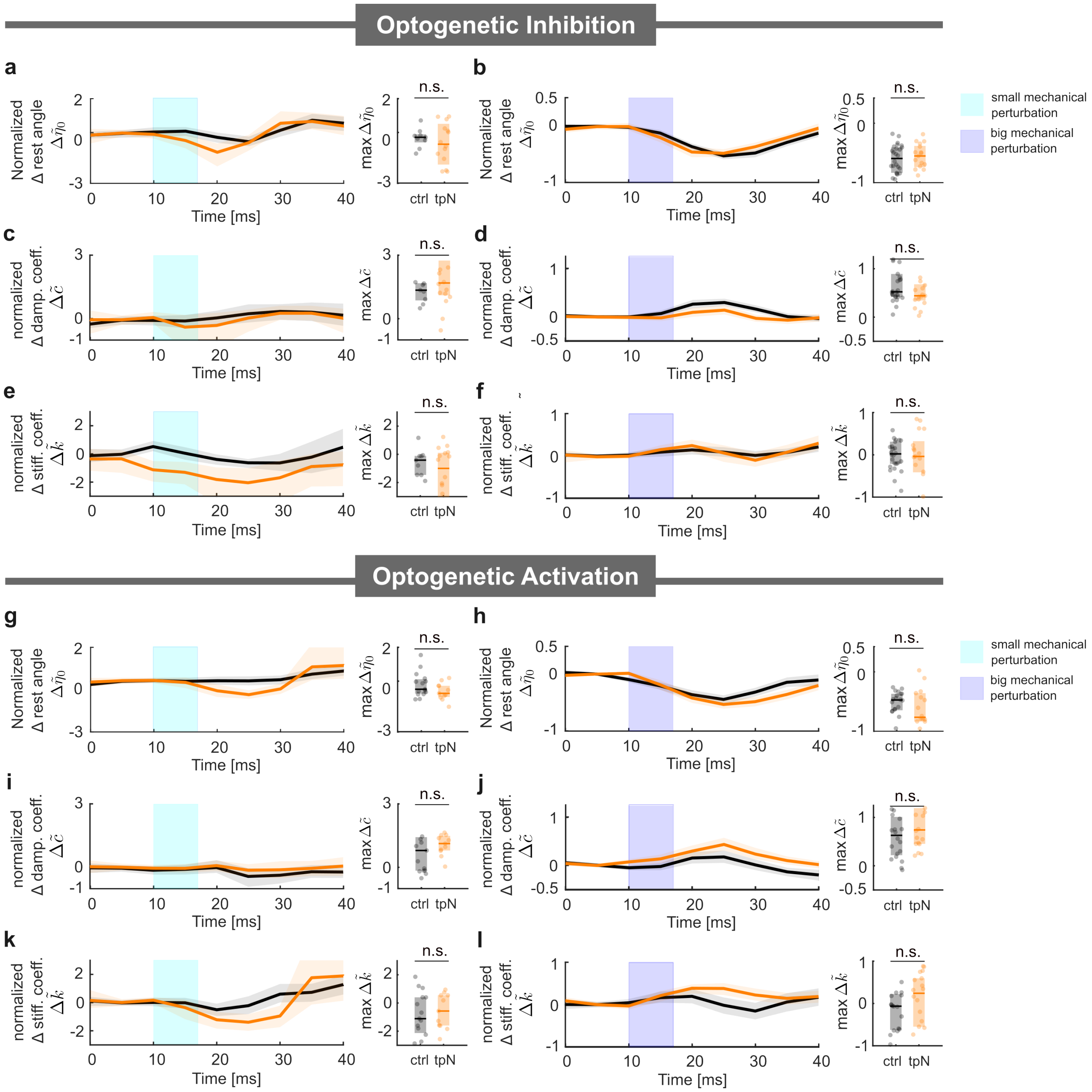

Figure S15. tpN torsional spring model fit (a,b) Left: Normalized change in the torsional spring rest angle: Comparison of control and tpN-inhibited flies in response to small and large pitch perturbations. Right: Maximum change in normalized torsional spring rest angle. (c,d) Left: Normalized change in the torsional spring damping factor: Comparison of control and tpN-inhibited flies in response to small and large pitch perturbations. Right: Maximum change in normalized torsional spring damping factor. (e,f) Left: Normalized change in the torsional spring stiffness constant: Comparison of control and tpN-inhibited flies in response to small and large pitch perturbations. Right: Maximum change in normalized torsional spring stiffness constant. (g,h) Left: Normalized change in the torsional spring rest angle: Comparison of control and tpN-activated flies in response to small and large pitch perturbations. Right: Maximum change in normalized torsional spring rest angle. (i,j) Left: Normalized change in the torsional spring damping factor: Comparison of control and tpN-activated flies in response to small and large pitch perturbations. Right: Maximum change in normalized torsional spring damping factor. (k,l) Left: Normalized change in the torsional spring stiffness constant: Comparison of control and tpN-activated flies in response to small and large pitch perturbations. Right: Maximum change in normalized torsional spring stiffness constant. Statistical significance for (a)-(l) is determined via the Kruskal-Wallis test with Dunn's post hoc multiple comparisons. (***, $p < .001$; **, $p < .01$; *, $p < .05$). Optogenetic inhibition small perturbation: control n=9, tpN n= 16. Optogenetic inhibition big perturbation: control n=26, tpN n= 17. Optogenetic activation small perturbation: control, n=15, tpN n= 11. Optogenetic activation big perturbation: control, n=19, tpN n= 17

bioRxiv preprint doi: https://doi.org/10.1101/2025.11.02.686144; this version posted November 8, 2025. The copyright holder for this preprint (which was not certified by peer review) is the author/funder, who has granted bioRxiv a license to display the preprint in perpetuity. It is made available under aCC-BY 4.0 International license.

Supplementary Note 5: Additional control theory analysis

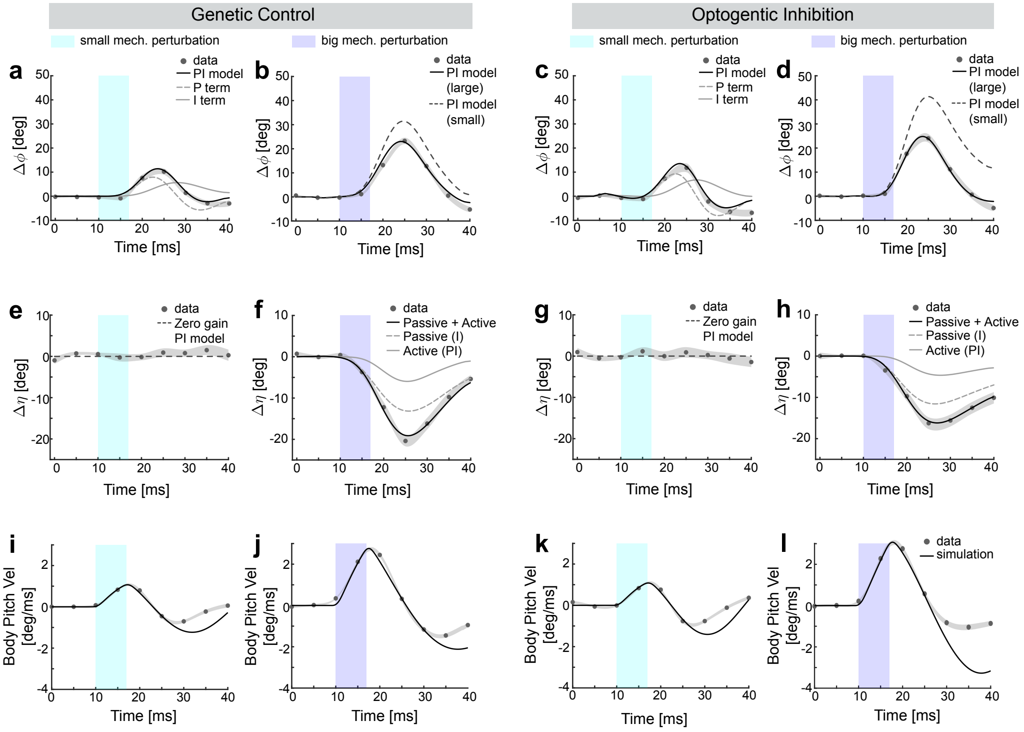

Figure S16. Control theoretic model fits for genetic control and tp1 inhibition data (a–d) Change in wing stroke angle over time for simulated flies (black lines, PI controller) fitted to the experimental data (black circles representing the population average, with the gray shaded region indicating the SEM) for small (a,c) and large (b,d) mechanical perturbation in the control (a, b) and tp1 inhibited (c, d) groups. Gray dashed and solid lines in (a,c) denote the contributions from the P term (proportional), and the I term (integral), respectively. The gray dashed line in (b,d) denotes the PI controller model fit with parameters from small perturbation. (e–h) Change in wing pitch angle over time for simulated flies (black lines) fitted to the experimental data (black circles representing the population average, with the gray shaded region indicating the SEM) for small (e,g) and big (f,h) perturbation in the control (e, f) and tp1 inhibited (g, h) groups. The dashed lines in (e,g) denote the zero gain PI controller model fit to the small perturbation data. The solid black line, dashed gray line, and solid gray line in (f,h) represent the combined passive and active controller model fit, the individual passive model fit, and the active model fit, respectively. (i–l) Change in body pitch velocity over time for simulated flies. The colors are same as in (a–h). Small perturbation: control (control) n=28, optogenetic inhibition (tp1 inh) n=27. Big perturbation: control n=61, tp1 inh n= 53.

bioRxiv preprint doi: https://doi.org/10.1101/2025.11.02.686144; this version posted November 8, 2025. The copyright holder for this preprint (which was not certified by peer review) is the author/funder, who has granted bioRxiv a license to display the preprint in perpetuity. It is made available under aCC-BY 4.0 International license.

	Stroke Controller				Pitch Controller Passive		Pitch Controller Active			
	K_p (/ms)		K_i [deg/deg]]		K_i [deg/deg]]		K_p (/ms)		K_i [(]deg/deg]	
	Small	Large	Small	Large	Small	Large	Small	Large	Small	Large
Control	7.5794±0.5503	6.2930±0.3821	0.6931±0.1028	0.3984±0.0472	0	-0.5±0.0998	0	-1.2±0.1499	0	-0.1616±0.0167
tp1 act	7.0000±1.0433	5.9261±0.1831	1.4±0.2250	0.5876±0.0266	-0.6280±0.0373	-0.65±0.0585	-0.8±0.2635	-1±0.3881	-0.0840±0.0562	-0.2489±0.0686
tp1 inh	8.6030±0.8889	7.0803±0.2982	0.7±0.1986	0.2299±0.0344	0	-0.4±0.0223	0	-0.5099±0.0738	0	-0.1472±0.0068
tp2 act	5.0806±0.7564	4.90173±0.3468	0.9286±0.1320	0.7478±0.0560	-0.5455±0.2541	-0.85±0.0393	-1±1.5922	-0.9±0.06426	-0.0840±0.2705	-0.1± 0.0945
tp2 inh	7.5801±0.3242	6.9967±0.2289	0.9178±0.0643	0.9379±0.0327	0	-0.3±0.0866	0	-0.6422±0.2795	0	-0.3497±0.0372
tpn act	7.9081±0.2926	5.1694±0.1242	0.4602±0.0384	0.5476±0.0166	0±0.0569	-0.85±0.0545	0±0.4495	-1±0.4981	0±0.0684	-0.1744±0.0755
tpn inh	7.5051±0.7256	5.5576±0.4266	0.8875±0.1515	0.7037±0.0702	0	-0.4±0.1368	0	-0.3092±0.1338	0	-0.2676±0.0164

Table 4. Fitted controller parameter values with mean and standard deviation.

bioRxiv preprint doi: https://doi.org/10.1101/2025.11.02.686144; this version posted November 8, 2025. The copyright holder for this preprint (which was not certified by peer review) is the author/funder, who has granted bioRxiv a license to display the preprint in perpetuity. It is made available under aCC-BY 4.0 International license.

Supplementary Note 6: Quasi-steady aerodynamic calculation parameter values

Variable	Definition	Value
m	fly body mass	1.34 mg
I_{pitch}	pitch moment of inertia	0.506 mgmm^2
$C_{friction}$	body pitch rotational drag	0.52 mgmm^2
R	wing span	2.5 mm
$\bar{c}$	mean wing chord length	0.7 mm
S	wing area	5.74 mm^2
$r_s^2(S)$	non-dimensionalized second moment of wing area	0.313
f	wingbeat frequency	225 Hz
r_{hinge}	distance from body center of mass to wing hinge	0.22 mm
$C_{\text{L}_{\max}}$	maximum lift coefficient	1.8
$C_{\text{D}_{\max}}$	maximum drag coefficient	3.4
C_{D_0}	minimum drag coefficient	0.4
C_{rot}	rotational force coefficient	1.57

Table 5. Body and wing morphological data.

bioRxiv preprint doi: https://doi.org/10.1101/2025.11.02.686144; this version posted November 8, 2025. The copyright holder for this preprint (which was not certified by peer review) is the author/funder, who has granted bioRxiv a license to display the preprint in perpetuity. It is made available under aCC-BY 4.0 International license.

887 # Supplementary Note 7: Full genotype of flies used in experiments

Data shown in	Experiment type	Abbreviated genotype	Full genotype
Figure 1 (c)	VNC anatomy	tp1-GAL4	w; 20XUAS-CsChrimson-mVenus (attP18)/+ ; 52E06.p65ADZp/+ ; 39G05.ZpGDBD.attp2/+
Figure 1 (c)	VNC anatomy	tp2-GAL4	w; 20XUAS-CsChrimson-mVenus (attP18)/+ ; VT021762.p65ADZp.attp40/+ ; VT042475.ZpGDBD.attp2/+
Figure 1 (c)	VNC anatomy	tpN-GAL4	w; 20XUAS-CsChrimson-mVenus (attP18)/73C04.p65ADZp.su(Hw)attP8 ; +/+ ; 60A06-ZpGDBD.attp2/+
Figure 1 (g and h)	opto. silencing (control) and pitch pert.	empty	w; UAS-GtACR1 (attP40)/R24A03-p65ADZp (attP40) ; R74C01-ZpGdbd (attP2)/+
Figure 1 (g and h)	opto. silencing and pitch pert.	tp1-GAL4	w; UAS-GtACR1 (attP40)/+ ; 52E06.p65ADZp/+ ; 39G05.ZpGDBD.attp2/+
Figure 1 (g and h)	opto. silencing and pitch pert.	tp1-SG	w; UAS-GtACR1 (attP40)/+ ; VT022025.p65ADZp.attp2/VT029310.ZpGAL4DBD.attp2 ; +/+
Figure 2 (a)-(j)	opto. silencing (control) and pitch pert.	empty	w; UAS-GtACR1 (attP40)/R24A03-p65ADZp (attP40) ; R74C01-ZpGdbd (attP2)/+
Figure 2 (i)-(j)	opto. activation (control) and pitch pert.	empty	w; 20XUAS-CsChrimson-mVenus (attP18)/+ ; R24A03- p65ADZp (attP40)/+ ; R74C01-ZpGdbd (attP2)/+
Figure 2 (i)-(j)	pitch pert.	WT	+
Figure 3 (a)-(e)	opto. silencing (control) and pitch pert.	empty	w; UAS-GtACR1 (attP40)/R24A03-p65ADZp (attP40) ; R74C01-ZpGdbd (attP2)/+
Figure 3 (a)-(e)	opto. silencing and pitch pert.	tp1-GAL4	w; UAS-GtACR1 (attP40)/+ ; 52E06.p65ADZp/+ ; 39G05.ZpGDBD.attp2/+
Figure 3 (a)-(e)	opto. silencing and pitch pert.	tp1-SG	w; UAS-GtACR1 (attP40)/+ ; VT022025.p65ADZp.attp2/VT029310.ZpGAL4DBD.attp2 ; +/+
Figure 3 (f)-(j)	opto. activation (control) and pitch pert.	empty	w; 20XUAS-CsChrimson-mVenus (attP18)/+ ; R24A03- p65ADZp (attP40)/+ ; R74C01-ZpGdbd (attP2)/+
Figure 3 (f)-(j)	opto. activation and pitch pert.	tp1-GAL4	w; 20XUAS-CsChrimson-mVenus (attP18)/+ ; 52E06.p65ADZp/+ ; 39G05.ZpGDBD.attp2/+
Figure 3 (f)-(j)	opto. activation and pitch pert.	tp1-SG	w; 20XUAS-CsChrimson-mVenus (attP18)/+ ;VT022025.p65ADZp.attp2/VT029310.ZpGAL4DBD.attp2 ; +/+
Figure 4 (a)-(i)	opto. silencing (control) and pitch pert.	empty	w; UAS-GtACR1 (attP40)/R24A03-p65ADZp (attP40) ; R74C01-ZpGdbd (attP2)/+
Figure 4 (g)-(i)	opto. activation (control) and pitch pert.	empty	w; 20XUAS-CsChrimson-mVenus (attP18)/+ ; R24A03- p65ADZp (attP40)/+ ; R74C01-ZpGdbd (attP2)/+
Figure 4 (g)-(i)	pitch pert.	WT	+
Figure 4 (p)-(r)	opto. silencing and pitch pert.	empty	w; UAS-GtACR1 (attP40)/R24A03-p65ADZp (attP40) ; R74C01-ZpGdbd (attP2)/+
Figure 4 (p)-(r)	opto. silencing and pitch pert.	tp1-GAL4	w; UAS-GtACR1 (attP40)/+ ; 52E06.p65ADZp/+ ; 39G05.ZpGDBD.attp2/+
Figure 4 (p)-(r)	opto. silencing and pitch pert.	tp1-SG	w; UAS-GtACR1 (attP40)/+ ; VT022025.p65ADZp.attp2/VT029310.ZpGAL4DBD.attp2 ; +/+
Figure 4 (s)-(u)	opto. activation (control) and pitch pert.	empty	w; 20XUAS-CsChrimson-mVenus (attP18)/+ ; R24A03- p65ADZp (attP40)/+ ; R74C01-ZpGdbd (attP2)/+
Figure 4 (s)-(u)	opto. activation and pitch pert.	tp1-GAL4	w; 20XUAS-CsChrimson-mVenus (attP18)/+ ; 52E06.p65ADZp/+ ; 39G05.ZpGDBD.attp2/+
Figure 4 (s)-(u)	opto. activation and pitch pert.	tp1-SG	w; 20XUAS-CsChrimson-mVenus (attP18)/+ ;VT022025.p65ADZp.attp2/VT029310.ZpGAL4DBD.attp2 ; +/+

Table 6. Full genotype of flies used in experiments (main text figures).

bioRxiv preprint doi: https://doi.org/10.1101/2025.11.02.686144; this version posted November 8, 2025. The copyright holder for this preprint (which was not certified by peer review) is the author/funder, who has granted bioRxiv a license to display the preprint in perpetuity. It is made available under aCC-BY 4.0 International license.

Data shown in	Experiment type	Abbreviated genotype	Full genotype
Figure **??** (a)-(d)	opto. silencing (control) and pitch pert.	empty	w; UAS-GtACR1 (attP40)/R24A03-p65ADZp (attP40) ; R74C01-ZpGdbd (attP2)/+
Figure S6 (a)-(b)	opto. silencing (control) and pitch pert.	empty	w; UAS-GtACR1 (attP40)/R24A03-p65ADZp (attP40) ; R74C01-ZpGdbd (attP2)/+
Figure S6 (a)-(b)	opto. activation (control) and pitch pert.	empty	w; 20XUAS-CsChrimson-mVenus (attP18)/+ ; R24A03- p65ADZp (attP40)/+ ; R74C01-ZpGdbd (attP2)/+
Figure S6 (a)-(b)	pitch pert.	empty	+
Figure **??** (a)-(c)	opto. silencing (control) and pitch pert.	empty	w; UAS-GtACR1 (attP40)/R24A03-p65ADZp (attP40) ; R74C01-ZpGdbd (attP2)/+
Figure **??** (a)-(c)	opto. activation (control) and pitch pert.	empty	w; 20XUAS-CsChrimson-mVenus (attP18)/+ ; R24A03- p65ADZp (attP40)/+ ; R74C01-ZpGdbd (attP2)/+
Figure **??** (a)-(c)	pitch pert.	empty	+
Figure **??** (c)	opto. silencing and pitch pert.	tp1-GAL4	w; UAS-GtACR1 (attP40)/+ ; 52E06.p65ADZp/+ ; 39G05.ZpGDBD.attp2/+
Figure **??** (c)	opto. silencing and pitch pert.	tp1-SG	w; UAS-GtACR1 (attP40)/+ ; VT022025.p65ADZp.attp2/VT029310.ZpGAL4DBD.attp2 ; +/+
Figure **??**(c)	opto. activation and pitch pert.	tp1-GAL4	w; 20XUAS-CsChrimson-mVenus (attP18)/+ ; 52E06.p65ADZp/+ ; 39G05.ZpGDBD.attp2/+
Figure **??**(c)	opto. activation and pitch pert.	tp1-SG	w; 20XUAS-CsChrimson-mVenus (attP18)/+ ; VT022025.p65ADZp.attp2/VT029310.ZpGAL4DBD.attp2 ; +/+
Figure S5(a)	CNS and muscle anatomy	tp1-SG	w; 20XUAS-CsChrimson-mVenus (attP18)/+ ; VT022025.p65ADZp.attp2/VT029310.ZpGAL4DBD.attp2 ; +/+
Figure S5(b)	Brain and muscle anatomy	tp1-GAL4	w; 20XUAS-CsChrimson-mVenus (attP18)/+ ; 52E06.p65ADZp/+ ; 39G05.ZpGDBD.attp2/+
Figure S5(c)	Brain and muscle anatomy	tp2-GAL4	w; 20XUAS-CsChrimson-mVenus (attP18)/+ ; VT021762.p65ADZp.attp40/+ ; VT042475.ZpGDBD.attp2/+
Figure S5(d)	Brain and muscle anatomy	tpN-GAL4	w; 20XUAS-CsChrimson-mVenus (attP18)/73C04.p65ADZp.su(Hw)attP8 ; +/+ ; 60A06-ZpGDBD.attp2/+
Figure S7 (a)-(c)	opto. activation (control) and pitch pert.	empty	w; 20XUAS-CsChrimson-mVenus (attP18)/R24A03-p65ADZp (attP40) ; R74C01-ZpGdbd (attP2)/+
Figure S7 (a)-(c)	opto. activation and pitch pert.	tp1-GAL4	w; 20XUAS-CsChrimson-mVenus (attP18)/+ ; 52E06.p65ADZp/+ ; 39G05.ZpGDBD.attp2/+
Figure S7 (a)-(c)	opto. activation and pitch pert.	tp1-SG	w; 20XUAS-CsChrimson-mVenus (attP18)/+ ; VT022025.p65ADZp.attp2/VT029310.ZpGAL4DBD.attp2 ; +/+
Figure S7(d)	opto. silencing (control) and pitch pert.	empty	w; UAS-GtACR1 (attP40)/R24A03-p65ADZp (attP40) ; R74C01-ZpGdbd (attP2)/+
Figure S7(d)	opto. silencing and pitch pert.	tp1-GAL4	w; UAS-GtACR1 (attP40)/+ ; 52E06.p65ADZp/+ ; 39G05.ZpGDBD.attp2/+
Figure S7(d)	opto. silencing and pitch pert.	tp1-SG	w; UAS-GtACR1 (attP40)/+ ; VT022025.p65ADZp.attp2/VT029310.ZpGAL4DBD.attp2 ; +/+
Figure S8 (a)-(c)	opto. activation (control) and pitch pert.	empty	w; 20XUAS-CsChrimson-mVenus (attP18)/R24A03-p65ADZp (attP40) ; R74C01-ZpGdbd (attP2)/+
Figure S8 (a)-(c)	opto. activation and pitch pert.	tp1-GAL4	w; 20XUAS-CsChrimson-mVenus (attP18)/+ ; 52E06.p65ADZp/+ ; 39G05.ZpGDBD.attp2/+
Figure S8 (a)-(c)	opto. activation and pitch pert.	tp1-SG	w; 20XUAS-CsChrimson-mVenus (attP18)/+ ; VT022025.p65ADZp.attp2/VT029310.ZpGAL4DBD.attp2 ; +/+
Figure S8 (d)-(f)	opto. silencing (control) and pitch pert.	empty	w; UAS-GtACR1 (attP40)/R24A03-p65ADZp (attP40) ; R74C01-ZpGdbd (attP2)/+
Figure S8 (d)-(f)	opto. silencing and pitch pert.	tp1-GAL4	w; UAS-GtACR1 (attP40)/+ ; 52E06.p65ADZp/+ ; 39G05.ZpGDBD.attp2/+
Figure S8 (d)-(f)	opto. silencing and pitch pert.	tp1-SG	w; UAS-GtACR1 (attP40)/+ ; VT022025.p65ADZp.attp2/VT029310.ZpGAL4DBD.attp2 ; +/+
Figure S9 (a)-(h)	opto. silencing (control) and pitch pert.	empty	w; UAS-GtACR1 (attP40)/R24A03-p65ADZp (attP40) ; R74C01-ZpGdbd (attP2)/+
Figure S9 (a)-(h)	opto. silencing and pitch pert.	tp1-GAL4	w; UAS-GtACR1 (attP40)/+ ; 52E06.p65ADZp/+ ; 39G05.ZpGDBD.attp2/+
Figure S9 (a)-(h)	opto. silencing and pitch pert.	tp1-SG	w; UAS-GtACR1 (attP40)/+ ; VT022025.p65ADZp.attp2/VT029310.ZpGAL4DBD.attp2 ; +/+
Figure S9 (i)-(p)	opto. activation (control) and pitch pert.	empty	w; 20XUAS-CsChrimson-mVenus (attP18)/R24A03-p65ADZp (attP40) ; R74C01-ZpGdbd (attP2)/+
Figure S9 (i)-(p)	opto. activation and pitch pert.	tp1-GAL4	w; 20XUAS-CsChrimson-mVenus (attP18)/+ ; 52E06.p65ADZp/+ ; 39G05.ZpGDBD.attp2/+
Figure S9 (i)-(p)	opto. activation and pitch pert.	tp1-SG	w; 20XUAS-CsChrimson-mVenus (attP18)/+ ; VT022025.p65ADZp.attp2/VT029310.ZpGAL4DBD.attp2 ; +/+
Figure S10 (a)-(h)	opto. silencing (control) and pitch pert.	empty	w; UAS-GtACR1 (attP40)/R24A03-p65ADZp (attP40) ; R74C01-ZpGdbd (attP2)/+
Figure S10 (a)-(h)	opto. silencing and pitch pert.	tp2-GAL4	w; UAS-GtACR1 (attP40)/+ ; VT021762.p65ADZp.attp40/+ ; VT042475.ZpGDBD.attp2/+
Figure S10 (i)-(p)	opto. activation (control) and pitch pert.	empty	w; 20XUAS-CsChrimson-mVenus (attP18)/R24A03-p65ADZp (attP40) ; R74C01-ZpGdbd (attP2)/+
Figure S10 (i)-(p)	opto. activation and pitch pert.	tp2-GAL4	w; 20XUAS-CsChrimson-mVenus (attP18)/+ ; VT021762.p65ADZp.attp40/+ ; VT042475.ZpGDBD.attp2/+
Figure S11 (a)-(h)	opto. silencing (control) and pitch pert.	empty	w; UAS-GtACR1 (attP40)/R24A03-p65ADZp (attP40) ; R74C01-ZpGdbd (attP2)/+
Figure S11 (a)-(h)	opto. silencing and pitch pert.	tpN-GAL4	w; UAS-GtACR1 (attP40)/73C04.p65ADZp.su(Hw)attP8 ; +/+ ; 60A06-ZpGDBD.attp2/+
Figure S11 (i)-(p)	opto. activation (control) and pitch pert.	empty	w; 20XUAS-CsChrimson-mVenus (attP18)/R24A03-p65ADZp (attP40) ; R74C01-ZpGdbd (attP2)/+
Figure S11 (i)-(p)	opto. activation and pitch pert.	tpN-GAL4	w; 20XUAS-CsChrimson-mVenus (attP18)/73C04.p65ADZp.su(Hw)attP8 ; +/+ ; 60A06-ZpGDBD.attp2/+
Figure S12 (a)-(h)	opto. silencing (control) and pitch pert.	empty	w; UAS-GtACR1 (attP40)/R24A03-p65ADZp (attP40) ; R74C01-ZpGdbd (attP2)/+
Figure S12 (a)-(h)	opto. silencing and pitch pert.	tp2-GAL4	w; UAS-GtACR1 (attP40)/+ ; VT021762.p65ADZp.attp40/+ ; VT042475.ZpGDBD.attp2/+
Figure S12 (i)-(p)	opto. activation (control) and pitch pert.	empty	w; 20XUAS-CsChrimson-mVenus (attP18)/R24A03-p65ADZp (attP40) ; R74C01-ZpGdbd (attP2)/+
Figure S12 (i)-(p)	opto. activation and pitch pert.	tp2-GAL4	w; 20XUAS-CsChrimson-mVenus (attP18)/+ ; VT021762.p65ADZp.attp40/+ ; VT042475.ZpGDBD.attp2/+
Figure S13 (a)-(f)	opto. silencing (control) and pitch pert.	empty	w; UAS-GtACR1 (attP40)/R24A03-p65ADZp (attP40) ; R74C01-ZpGdbd (attP2)/+
Figure S13 (a)-(f)	opto. silencing and pitch pert.	tp2-GAL4	w; UAS-GtACR1 (attP40)/+ ; VT021762.p65ADZp.attp40/+ ; VT042475.ZpGDBD.attp2/+
Figure S13 (g)-(l)	opto. activation (control) and pitch pert.	empty	w; 20XUAS-CsChrimson-mVenus (attP18)/R24A03-p65ADZp (attP40) ; R74C01-ZpGdbd (attP2)/+
Figure S13 (g)-(l)	opto. activation and pitch pert.	tp2-GAL4	w; 20XUAS-CsChrimson-mVenus (attP18)/+ ; VT021762.p65ADZp.attp40/+ ; VT042475.ZpGDBD.attp2/+
Figure S14 (a)-(h)	opto. silencing (control) and pitch pert.	empty	w; UAS-GtACR1 (attP40)/R24A03-p65ADZp (attP40) ; R74C01-ZpGdbd (attP2)/+
Figure S14 (a)-(h)	opto. silencing and pitch pert.	tpN-GAL4	w; UAS-GtACR1 (attP40)/73C04.p65ADZp.su(Hw)attP8 ; +/+ ; 60A06-ZpGDBD.attp2/+
Figure S14 (i)-(p)	opto. activation (control) and pitch pert.	empty	w; 20XUAS-CsChrimson-mVenus (attP18)/R24A03-p65ADZp (attP40) ; R74C01-ZpGdbd (attP2)/+
Figure S14 (i)-(p)	opto. activation and pitch pert.	tpN-GAL4	w; 20XUAS-CsChrimson-mVenus (attP18)/73C04.p65ADZp.su(Hw)attP8 ; +/+ ; 60A06-ZpGDBD.attp2/+
Figure S15 (a)-(f)	opto. silencing (control) and pitch pert.	empty	w; UAS-GtACR1 (attP40)/R24A03-p65ADZp (attP40) ; R74C01-ZpGdbd (attP2)/+
Figure S15 (a)-(f)	opto. silencing and pitch pert.	tpN-GAL4	w; UAS-GtACR1 (attP40)/73C04.p65ADZp.su(Hw)attP8 ; +/+ ; 60A06-ZpGDBD.attp2/+
Figure S15 (g)-(l)	opto. activation (control) and pitch pert.	empty	w; 20XUAS-CsChrimson-mVenus (attP18)/R24A03-p65ADZp (attP40) ; R74C01-ZpGdbd (attP2)/+
Figure S15 (g)-(l)	opto. activation and pitch pert.	tpN-GAL4	w; 20XUAS-CsChrimson-mVenus (attP18)/73C04.p65ADZp.su(Hw)attP8 ; +/+ ; 60A06-ZpGDBD.attp2/+

Table 7. Full genotype of flies used in experiments (SI figures).
